# Let-7e modulates the inflammatory response in vascular endothelial cells through ceRNA crosstalk

**DOI:** 10.1038/srep42498

**Published:** 2017-02-14

**Authors:** Zongwei Lin, Junfeng Ge, Zhe Wang, Jianwei Ren, Xiaowei Wang, Hui Xiong, Jing Gao, Yan Zhang, Qunye Zhang

**Affiliations:** 1 The Key Laboratory of Cardiovascular Remodeling and Function Research, Chinese Ministry of Education and Ministry of Public Health; The State and Shandong Province Joint Key Laboratory of Translational Cardiovascular Medicine; Qilu Hospital, Shandong University, Jinan, China; 2 The Second People’s Hospital of Jinan, Jinan, China; 3 Division of Endocrinology and Metabolism, Shandong Provincial Hospital affiliated to Shandong University, Jinan, China; 4 Health Division of Guard Bureau, General Staff Department of Chinese PLA, Beijing, China; 5 Shandong Cancer Hospital Affiliated to Shandong University, Shandong Academy of Medical Sciences, Jinan, China; 6 Department of Pharmacology, Shandong University School of Medicine, Jinan, China

## Abstract

The inflammatory responses of vascular endothelial cells (VECs) are critical in the development of many cardio-cerebrovascular diseases. Let-7e is an important regulator of endothelial function and inflammation. However, the effects and mechanisms of let-7e on VECs inflammation have not been studied until recently. Thus, we investigated these issues and found that in addition to proliferation, apoptosis and cell adhesion, let-7e was also implicated in the regulation of inflammatory responses through a complex network, including IκBβ and lncRNA lnc-MKI67IP-3. Let-7e promoted NF-κB activation and translocation to the nucleus by inhibiting its target gene (IκBβ) expression and subsequently increased the expression of inflammatory and adhesion molecules. Meanwhile, lnc-MKI67IP-3 acted as a sponge or competing endogenous RNA (ceRNA) for let-7e, suppressing its pro-inflammatory effects, and let-7e decreased lnc-MKI67IP-3 expression, thereby forming a positive feedback loop to aggravate inflammation. Moreover, let-7e, lnc-MKI67IP-3 and IκBβ were also abnormal in oxLDL-treated VECs and atherosclerotic plaques. The present study revealed let-7e as a pro-inflammatory mediator and a novel regulatory mechanism for the NF-κB pathway through ceRNA crosstalk, comprising let-7e and its target IκBβ and the ceRNA lnc-MKI67IP-3. Thus, this molecule might play important roles in the inflammatory responses of VECs and development of atherosclerosis.

Numerous studies have demonstrated that vascular endothelial cells (ECs) are crucial in the regulation of immune and inflammatory responses^1^. In ECs activated by inflammatory stimuli, many adhesion molecules and pro-inflammatory cytokines are up-regulated (such as ICAM1, E-selectin and IL-6) with the suppression of anti-inflammatory cytokines. These molecules promote the lymphocytes, monocytes and other immune cells to adhere and migrate through the vessel wall into inflammatory sites^1^,^2^. After the elimination of inflammatory stimuli, pro- and anti-inflammatory factors are rebalanced and ECs return to the resting state. The endothelial dysfunction resulting from excessive and/or prolonged inflammation is one of the most important initiating events in the development of atherosclerosis and many other cardio-cerebrovascular diseases^3^. Many cells and molecules are involved in the inflammatory response of ECs. Therefore, its regulation mechanisms are extremely complex and have not been fully elucidated thus far, and more novel regulators should be identified.

MiRNAs are endogenous non-coding RNAs of approximately 22 nucleotides that regulate gene expression through binding to the 3′ untranslated regions (UTRs) of their targets. Many studies have demonstrated miRNAs as key regulators of inflammation in ECs. For example, miR-146a and miR-10a can repress inflammation by targeting several components (TRAF6/IRAK4) of the NF-κB pathway in ECs^4^. MiR-92a promotes inflammation in ECs by targeting KLF2/KLF4, which inhibits the expression of NF-κB dependent vascular inflammatory genes^5^. Additionally, miRNAs also directly target various adhesion molecules, playing anti-inflammatory roles in activated ECs, i.e., miR-31 targets SELE and miR-17-3p targets ICAM1^6^,^7^. Moreover, numerous miRNAs involved in endothelial inflammation play critical roles in many major diseases, such as atherosclerosis and diabetes^8^,^9^. However because of the complexity of inflammatory genes and miRNAs, there would be lots of undiscovered miRNAs associated with endothelial inflammation. Therefore, further studies are needed to reveal more miRNAs and their associated mechanisms underlying the inflammatory response of endothelial cells.

The let-7 family is evolutionarily conserved from bacteria to humans and can act as anti-inflammatory factors by targeting some pro-inflammatory factors (e.g., let-7d targets IL-13; let-7a targets IL-6)^10^,^11^, except for development and tumor suppression. These molecules can also act as pro-inflammatory factors by targeting some anti-inflammatory factors, including IL-10^12^. In addition to the direct pro-inflammatory effects, let-7f can also indirectly play pro-inflammatory roles by targeting A20, a feedback inhibitor of the NF-κB pathway in macrophages^13^. Additionally, the let-7 family is closely associated with endothelial function, including apoptosis, angiogenesis and the endothelial-to-mesenchymal transition^14^,^15^. Let-7e is a member of the let-7 family and also a key regulator of endothelial function and inflammation. For example, let-7e regulates the migration and tube formation of endothelial progenitor cells via targeting FASLG^16^. Let-7e overexpression can activate Th1 and Th17 cells and aggravate experimental autoimmune encephalomyelitis and Hashimoto’s disease by targeting IL-10^17^,^18^. Furthermore, let-7e expression is significantly increased in many cardiovascular diseases, including coronary heart disease^19^. Therefore, we speculated that let-7e might perform critical roles in the regulation of the inflammatory responses of endothelial cells by directly or indirectly targeting certain inflammatory genes. However, studies of this hypothesis have not been reported until recently.

In the present study, we investigated the actions of let-7e in human umbilical vein endothelial cells (HUVECs) and found that let-7e could exacerbate the inflammatory responses of ECs by promoting NF-κB translocation into the nucleus and activation through ceRNA crosstalk, which was composed of lnc-MKI67IP-3, let-7e and its target IκBβ.

## Results

### Enrichment analysis and expression profiles of mRNAs/lncRNAs regulated by let-7e

**Figure 1:**
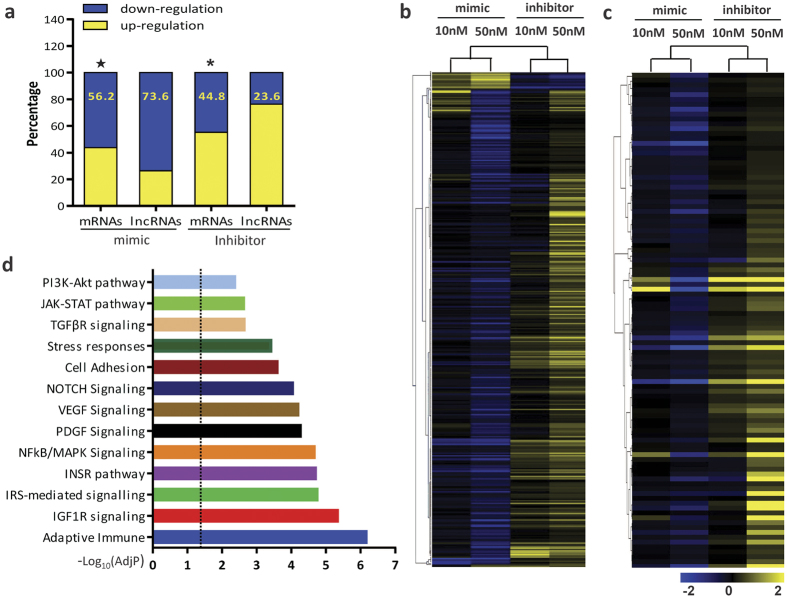
Expression profiles and enrichment Analysis of mRNAs/lncRNAs regulated by let-7e. **(a)** The percentage of down-regulated mRNAs (lncRNAs) in all regulated mRNAs (lncRNAs) in HUVECs treated by let-7e mimic or inhibitor. ^★^p < 0.05 vs. the percentage of down-regulated lncRNAs in HUVECs treated with let-7e mimic. *p < 0.05 vs. the percentage of down-regulated lncRNAs in HUVECs treated by let-7e inhibitor. **(b)** 385 mRNAs potentially targeted by let-7e and **(c)** 102 lncRNAs potentially binding to let-7e in HUVECs were hierarchically clustered. The effects of let-7e mimic and inhibitor (50 nM each) were obvious. The effects of let-7e mimic were predominantly inhibitory, while the effects of let-7e inhibitor were permissive. **(d)** The gene set enrichment analysis of the above-mentioned 487 transcripts was performed as described in the Methods section. Multiple testing was adjusted using the Benjamini–Hochberg correction. AdjP: adjusted P-value. The dotted line showed −log_10_ (0.05).

Let-7e was abundantly expressed in endothelial cells at an expression level higher (86%) than that of U6. The Ct value of let-7e relative to U6 was −0.9. As an internal control, U6 expression was also confirmed in HUVECs through different treatments (Supplementary Figure S1a). To determine the effects of let-7e on the expression patterns of mRNAs/lncRNAs in ECs, HUVECs were transfected with a let-7e inhibitor/mimic at different concentrations. The overexpression and underexpression of let-7e and the expression changes of other let-7 family members were verified (Supplementary Figure S1b,c). The results of the microarray analysis showed that let-7e profoundly affected the expression of mRNAs and lncRNAs (Fig. 1a). As described in Methods section, 385 mRNAs potentially targeted by let-7e and 102 lncRNAs potentially binding to let-7e were selected and hierarchically clustered (Fig. 1b and c). The gene set enrichment analysis of these 487 transcripts showed that let-7e was involved in many important biological processes and pathways, such as immune, inflammatory responses, cell proliferation and apoptosis, cell adhesion, and the VEGF and NF-κB/MAPK pathways (Fig. 1d).

### Co-expression/correlation network potentially targeted by let-7e

**Figure 2:**
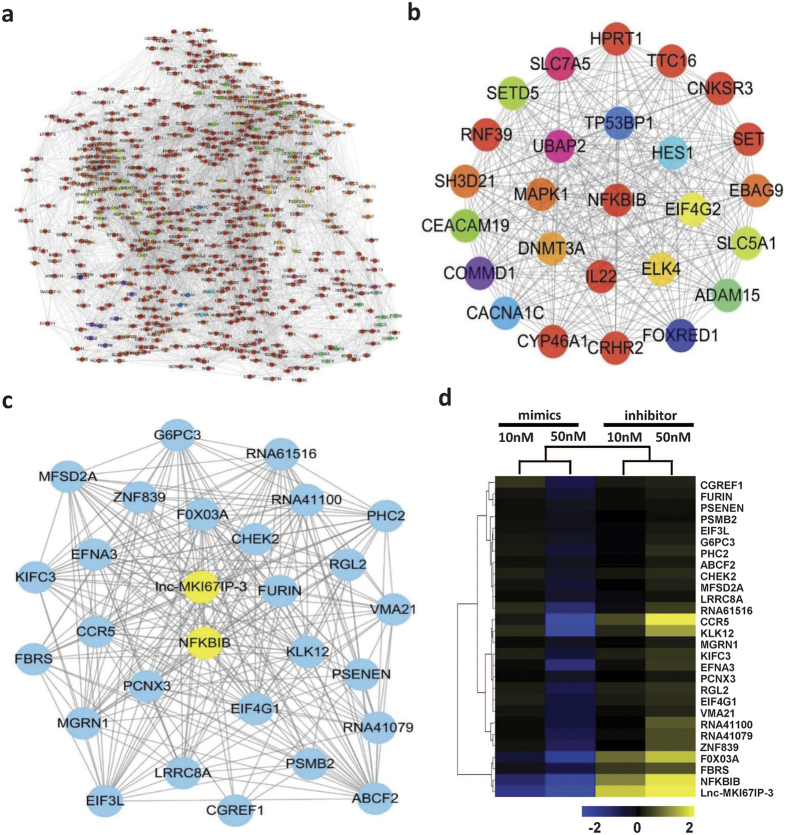
The co-expression/correlation network potentially targeted by let-7e. **(a)** The co-expression/correlation network of the aforementioned 487 transcripts was constructed as described in the Methods section. The lines between nodes represented significant co-expression/correlations (|r| > 0.99 and p < 0.05). **(b)** The shortlisting network comprising core nodes extracted from the whole network using ModuLand. The color of a core node represented a cluster of nodes in the entire network. **(c)** The core subnetwork was extracted from the whole network using MCODE, comprising transcripts significantly co-expressed with IκBβ (NFKBIB). **(d)** The expression data of the nodes constituting this network presented in (**c**) in different groups were hierarchically clustered. IκBβ and lnc-MKI67IP-3, the most connected nodes, were highlighted and their expression changes were most notable in these nodes.

The co-expression/correlation network was constructed based on the expression data from the above-mentioned 487 transcripts. Their correlations were extremely complex and constituted a large and complex co-expression/correlation network with >4100 edges (Fig. 2a). Then, the core nodes and subnetworks were selected as described in Methods. In the core subnetwork, most nodes were significantly co-expressed with IκBβ (|r| > 0.99 and p < 0.01) (Fig. 2b and c). Let-7e significantly down-regulated the expression of these molecules, while let-7e inhibitor had the opposite effects. Among these nodes, IκBβ and lnc-MKI67IP-3 were the most significantly regulated and their network connectivity, correlation coefficients (p value) and binding energies to let-7e were also higher than most other nodes (Fig. 2d, Supplementary Table S1). The expression of some nodes (CCR5 and FOXO3A) was also compared to that of IκBβ and lnc-MKI67IP-3 in HUVECs (Supplementary Figure S1d).

### Pro-inflammatory roles of let-7e in the inflammation of HUVECs

**Figure 3:**
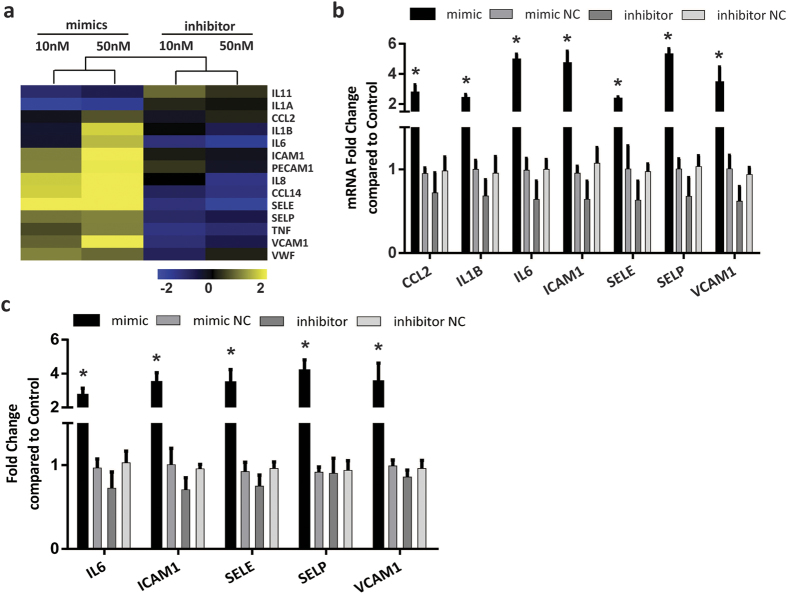
Inflammatory cytokines and adhesion molecules regulated by let-7e in HUVECs. **(a)** The expression data of important inflammation-associated genes in the aforesaid 487 transcripts were hierarchically clustered and presented. **(b)** and **(c)** HUVECs were treated with let-7e mimic or inhibitor and corresponding NC (50 nM each). Then, the mRNA expression of seven critical inflammatory cytokines and adhesion molecules was verified using qPCR (**b**). The secretion of five molecules was assayed using ELISA (**c**). The untreated HUVECs were used as control. *P < 0.05 vs. mimic/inhibitor NC and let-7e inhibitor groups. All experiments were repeated five times independently.

The microarray data suggested that let-7e could up-regulate the expression of many crucial pro-inflammatory cytokines and adhesion molecules in HUVECs, such as IL-1B, IL-6, ICAM1, VCAM1, SELE and SELP, while let-7e inhibitor down-regulated these molecules (Fig. 3a). Moreover, most of these molecules were regulated through NF-κB. Several important inflammatory cytokines and adhesion molecules were further verified using qPCR and ELISA. The results showed that let-7e mimic could significantly increase their secretion and mRNA expression, while the effects of let-7e inhibitor were not significant (Fig. 3b and c). These results might reflect the fact that the NF-κB pathway was inactive and inflammatory cytokines and adhesion molecules were expressed at low basal level in resting endothelial cells, and let-7e inhibitor treatment was difficult to further significantly decrease their expression.

### Let-7e exerted pro-inflammatory effects through targeting IκBβ and promoting NF-κB translocation

**Figure 4:**
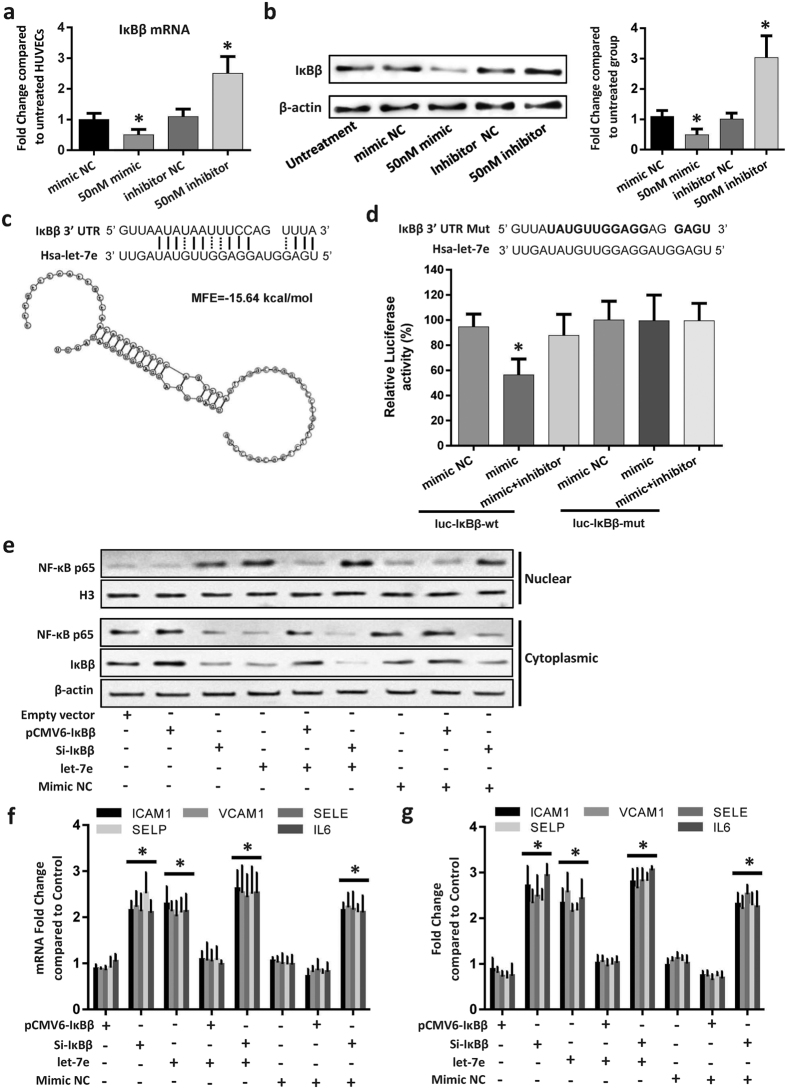
Let-7e played pro-inflammatory roles by promoting NF-κB translocation through targeting IκBβ. HUVECs were transfected alone or co-transfected with different combinations as shown in the figure for 24 hours. Subsequently, various experiments were performed. The expression of IκBβ **(a)** mRNA and **(b)** protein was detected. *P < 0.05 vs. all other groups. Full-length blots are presented in Supplementary Figure S3. **(c)** The predicted core binding sequence of let-7e in the 3′-UTR of IκBβ. The solid line represents standard base pairing and strong interactions; and the dashed line represents non-standard base pairing and relatively weak interactions. **(d)** Upper panel: the sequence alignment between let-7e and 3′-UTR mutant of IκBβ. The bold letters indicated the mutated bases. Lower panel: the luciferase activity in each group. HUVECs transfected with non-insert reporter (empty) vector were used as control. *P < 0.05 vs. all other groups. **(e)** IκBβ protein in the cytoplasm and NF-κB p65 protein in the cytoplasm and nucleus were detected by Western blotting. Let-7e promoted the nuclear translocation of NF-κB p65 by down-regulating its target gene IκBβ. Full-length blots are presented in Supplementary Figure S4. The total RNA from each group was purified, and the culture medium was also collected. Subsequently, the **(f)** mRNA expression and **(g)** protein secretion of five important inflammatory cytokines and adhesion molecules, transcriptionally regulated by NF-κB p65, were detected using qPCR and ELISA. HUVECs transfected with empty vector were used as controls. *p < 0.05 vs. pCMV6-IκBβ group, pCMV6-IκBβ + let-7e group, mimic NC group, and mimic NC + pCMV6-IκBβ group. All experiments were replicated five times independently.

The above results implied that IκBβ might play crucial roles in the pro-inflammatory effects of let-7e. Moreover, let-7e mimic significantly reduced IκBβ expression, but let-7e inhibitor showed the opposite effects (Fig. 4a and b). The minimum free energy (MFE) between let-7e and the predicted binding site of let-7e in IκBβ 3′ UTR was −15.6 kcal/mol (Fig. 4c), a value much lower than the experimentally confirmed MFEs between many miRNAs and their targets. For example, the MFE between miR-21 and its target PDCD4 is only −10 kcal/mol^20^. The luciferase reporter assay also showed that let-7e mimic, but not the mimic negative control, significantly reduced the relative luciferase activity in HUVECs transfected with Luc-IκBβ-WT. The luciferase activity in HUVECs transfected with Luc-IκBβ-Mut did not significantly change (Fig. 4d). These results indicated that IκBβ was the target of let-7e in HUVECs. Immunoblotting and cell immunofluorescence showed that let-7e significantly increased the nuclear translocation of NF-κB. IκBβ siRNA and/or let-7e had the same effects and also significantly down-regulated IκBβ (Fig. 4e, Supplementary Figure S1e). Moreover, compared with the control group, IκBβ siRNA and let-7e mimic significantly increased the expression and secretion of several important inflammation-associated molecules regulated by NF-κB. The IκBβ expression vector attenuated the above effects of let-7e mimic, and IκBβ siRNA further reinforced these effects (Fig. 4e–g). The primary function of IκBβ was to sequester NF-κB in the cytosol and inhibit the NF-κB pathway. In resting endothelial cells, NF-κB was primarily inactivated and almost all NF-κB were sequestered in the cytosol. Even IκBβ expression was further increased, and no additional NF-κB could be arrested in the cytoplasm and inhibited. Therefore, IκBβ expression vector treatment alone or combined with let-7e mimic NC had no significant effect on the nuclear translocation of NF-κB and expression/secretion of several inflammation-associated molecules (Fig. 4e–g).

### Lnc-MKI67IP-3 inhibited the pro-inflammatory effects of let-7e by functioning as a ceRNA

**Figure 5:**
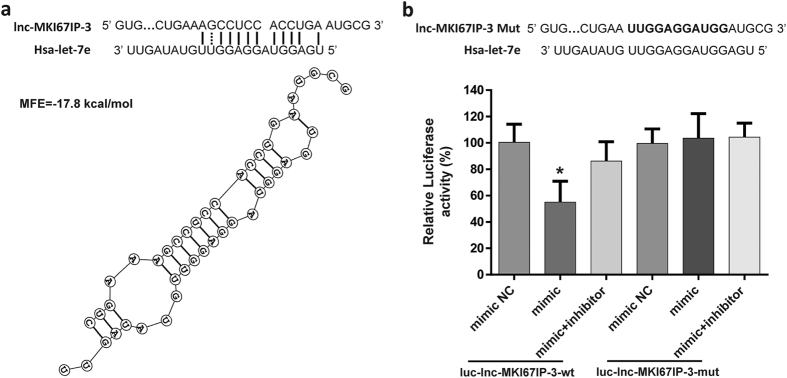
Lnc-MKI67IP-3 could bind to let-7e as a miRNA sponge . **(a)** The predicted binding sequence of let-7e in lnc-MKI67IP-3 sequence. The solid line represents standard base pairing and strong interactions; and the dashed line represents non-standard base pairing and relatively weak interactions. **(b)** Upper panel: the sequence alignment between let-7e and mutant of let-7e binding sequence in lnc-MKI67IP-3. The bold letters indicated the mutated sequence. Lower panel: HUVECs were transfected alone or co-transfected with different combinations as shown in the figure for 24 hours. Then, the luciferase activity in each group was assayed. HUVECs transfected with non-insert reporter (empty) vector were used as control. *p < 0.05 vs. all other groups. All experiments were repeated five times independently.

**Figure 6:**
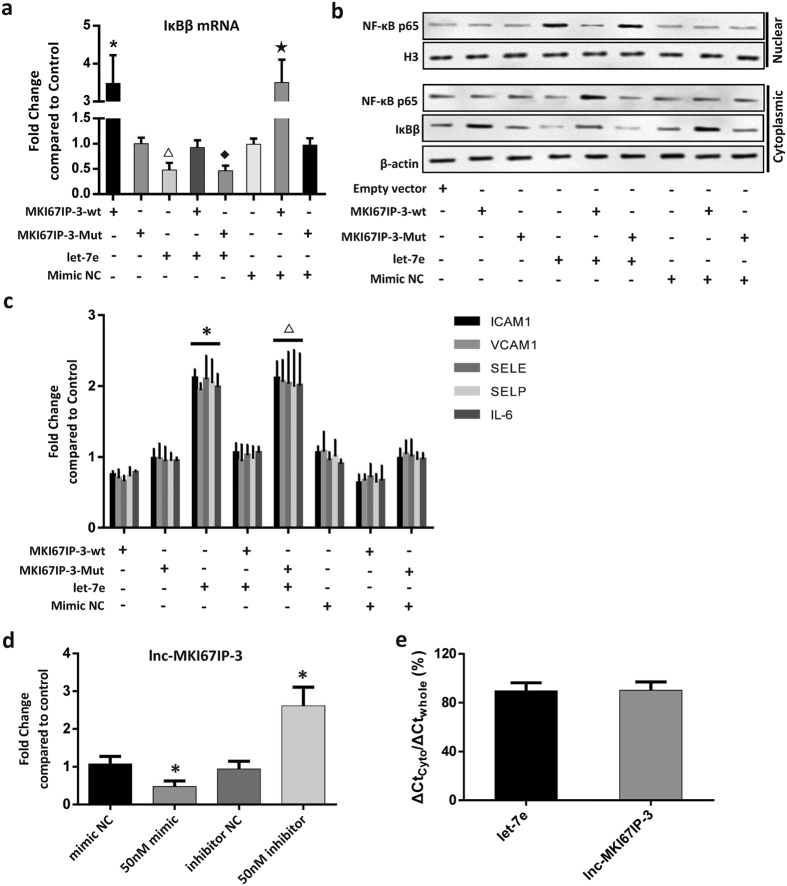
Lnc-MKI67IP-3 inhibited the pro-inflammatory effects of let-7e as a ceRNA. HUVECs were respectively treated as shown in the figure for 24 hours. Subsequently, various assays were performed. **(a)** The mRNA expression of IκBβ was assayed by qPCR. HUVECs transfected with empty vector were used as controls. *P < 0.05 vs. all other groups except for the MKI67IP-3-wt + mimic NC group; **^△^**P < 0.05 vs. all other groups except for the let-7e + MKI67IP-3-mut (lacking the binding site of let-7e) group; **^†^**P < 0.05 vs. all other groups except for let-7e group; ^**★**^P < 0.05 vs. all other groups except for MKI67IP-3-wt group. **(b)** IκBβ protein in the cytoplasm and NF-κB p65 protein in the cytoplasm and nucleus was detected using Western blotting. The results showed that lnc-MKI67IP-3 could antagonize the effects of let-7e on the IκBβ expression and nuclear translocation of NF-κB p65. Full-length blots are presented in the Supplementary Figure S5. **(c**) In the culture medium of each group, the secretion of five important inflammatory cytokines and adhesion molecules, transcriptionally regulated through NF-κB p65, was detected using ELISA. HUVECs transfected with empty vector were used as controls. *p < 0.05 vs. all other groups except for let-7e+ MKI67IP-3-Mut group. ****^△^p < 0.05 vs. all other groups except for let-7e group. **(d)** Lnc-MKI67IP-3 expression in HUVECs with different treatments was detected using qPCR. The untreated HUVECs were used as controls. *P < 0.05 vs. all other groups. **(e)** The expression of mature let-7e and lnc-MKI67IP-3 in the cytoplasm (cyto) and whole HUVECs (whole) were detected using qPCR.

Lnc-MKI67IP-3 is located on chromosome 2 and its function has not been described thus far. The present study showed a strong correlation between lnc-MKI67IP-3 and let-7e or IκBβ. The predicted results indicated that let-7e could bind to lnc-MKI67IP-3. The MFE between let-7e and the let-7e binding site in lnc-MKI67IP-3 was −17.8 kcal/mol (Fig. 5a). Luciferase reporter assay showed that only let-7e mimic significantly reduced the relative luciferase activity in HUVECs transfected with luc-lnc-MKI67IP-3-wt. The luciferase activity in other groups did not significantly change (Fig. 5b). This finding indicated that lnc-MKI67IP-3 could bind to let-7e and might act as a sponge to suppress the inhibitory effects of let-7e on its targets. Compared with the control group (transfection with empty vector), the lnc-MKI67IP-3-wt vector attenuated the let-7e-mediated inhibition of IκBβ expression and significantly increase the expression of IκBβ mRNA and protein. The lnc-MKI67IP-3-wt vector also attenuated the let-7e-mediated the nuclear translocation of NF-κB and secretion of numerous inflammatory molecules regulated through NF-κB (Fig. 6a–c). However, for the similar reasons mentioned in above section, lnc-MKI67IP-3-wt vector treatment alone or combined with let-7e mimic NC had no significant effect on the nuclear translocation of NF-κB and secretion of several inflammatory molecules. Furthermore, the lnc-MKI67IP-3-mut vector (lacking let-7e binding site) and the mimic NC also had no significant effects (Fig. 6a–c). Moreover, let-7e mimic significantly down-regulated lnc-MKI67IP-3 expression, while let-7e inhibitor markedly up-regulated this expression and let-7e inhibitor could be considered as an inducer of lnc-MKI67IP-3, just like vitamin C (Fig. 6d, Supplementary Figure S2a). At high concentration, let-7e inhibitor could completely reverse the effects of let-7e, namely increasing the expression of lnc-MKI67IP-3 and IkBβ (Supplementary Figure S2b and c). Lnc-MKI67IP-3 and mature let-7e were primarily localized in the cytoplasm (Fig. 6e), suggesting that lnc-MKI67IP-3 could inhibit the pro-inflammatory effects of let-7e as a ceRNA through binding to let-7e in the cytoplasm.

### Let-7e was up-regulated in ox-LDL-treated HUVECs and atherosclerotic plaques

**Figure 7:**
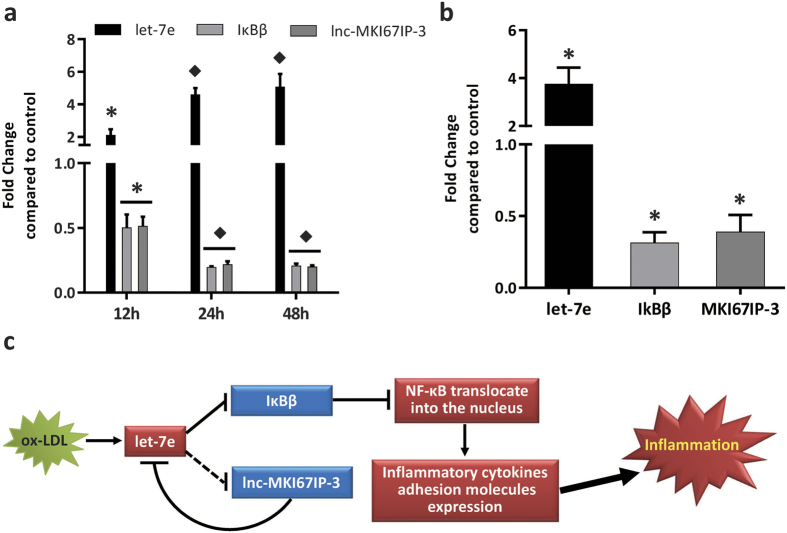
The ceRNA network comprising let-7e, IκBβ and lnc-MKI67IP-3 was abnormal in HUVECs treated with ox-LDL and atherosclerotic plaques. (**a**) HUVECs were treated with ox-LDL (50 μg/ml) for 12, 24 and 48 hours. The expression of let-7e, lnc-MKI67IP-3 and IkBβ was detected using qPCR. Untreated HUVECs were used as controls. *P < 0.05 vs. all other groups. ^†^P < 0.05 vs. control and 12 hours groups. (**b**) The expression of let-7e, IkBβ and lnc-MKI67IP-3 in human atherosclerotic plaques was detected using qPCR. Normal large arteries were used as controls. *P < 0.05 vs. control group. Experiments were repeated three times independently. (**c**) The schematic diagram of the pro-inflammatory effects of let-7e via ceRNA crosstalk in vascular endothelial cells. The red color indicates up-regulation, while the blue color indicates down-regulation. The solid line represents direct regulation, while the dotted line represents an unknown/indirect regulation.

Because of the instability of ox-LDL, its oxidation was confirmed prior to experiments (Supplementary Figure S6a). In HUVECs treated with ox-LDL for different times, ox-LDL significantly up-regulated let-7e and pri-let-7e expression, while the expression levels of IκBβ and lnc-MKI67IP-3 were significantly down-regulated (Fig. 7a, Supplementary Figure S6b). Although the effects of let-7e on apoptosis and proliferation were slight in HUVECs without ox-LDL treatment, let-7e mimic significantly further increased the apoptosis, proliferation inhibition, endothelial inflammation and monocyte-endothelial cell adhesion induced by ox-LDL in HUVECs. However, the opposite effects of let-7e inhibitor were observed (Supplementary Figure S6c–f and S7a–e). Moreover, the expression of let-7e, IκBβ and lnc-MKI67IP-3 in human atherosclerosis plaques was similar to that in oxLDL-treated HUVECs (Fig. 7b). These results indicated that the pro-inflammatory effects of let-7e via ceRNA crosstalk among lnc-MKI67IP-3, let-7e and IκBβ might play important roles in the inflammatory responses of endothelial cells induced by ox-LDL and atherosclerosis development.

## Discussion

Noncoding transcripts (>200 nucleotides) are defined as lncRNAs. To date, thousands of putative lncRNAs have been described. However, the precise functions and mechanisms of these molecules remain elusive. Accumulated studies have demonstrated that lncRNAs could act as crucial regulators in various pathophysiological processes. Particularly, some lncRNAs play essential roles in the regulation of endothelial cell function and inflammation. For example, some vascular-enriched lncRNAs, such as SENCR, MIR503HG, and HIF1A-AS1, regulated the proliferation, apoptosis, and angiogenesis of HUVECs^21^,^22^,^23^. LncRNA MALAT1 could up-regulate pro-inflammatory mediators (IL-6 and TNF-a) through SAA3 activation^24^. TUG1 could inhibit inflammatory responses as an anti-inflammatory mediator^25^. The present study revealed that lnc-MKI67IP-3 could also suppress let-7e-mediated inflammation in ECs. These findings suggest that lncRNA is a new class of inflammatory regulators in ECs, and more inflammation-associated lncRNAs should be discovered.

Many lncRNAs have miRNA recognition elements specific to certain miRNAs and can competitively inhibit the binding of miRNAs to their target genes, similar to miRNA sponges, and thus suppress their functions. This crosstalk between lncRNAs and miRNAs is called the ‘competing endogenous RNAs (ceRNAs)’ network and has been supported by numerous studies^26^. Many lncRNAs, acting as ceRNAs, have been identified, such as linc-MD1, H19, and lncRNA-BGL3^27^,^28^,^29^. However, studies on how ceRNAs regulate endothelial function are still in their infancy. MALAT1 acts as a ceRNA that competes with miR-22–3p and protects the endothelium from ox-LDL-induced dysfunction^30^. MIAT sequesters miR-150-5p as a ceRNA and alleviates its repressive effect on VEGF expression, thereby regulating the proliferation, migration, and survival of endothelial cells^31^. TGFB2-OT1 is involved in the autophagy and inflammation of vascular endothelial cells as a ceRNA by binding to miR-3960, miR-4488 and miR-4459, which targets CERS1, NAT8L, and LARP1, respectively^32^. The present study revealed that lnc-MKI67IP-3 antagonized the inhibitory effect of let-7e on its target IκBβ as a let-7e sponge, forming a ceRNA network comprising lnc-MKI67IP-3, let-7e and IκBβ. Meanwhile, let-7e could down-regulate lnc-MKI67IP-3, enhancing the suppressive effect of let-7e on IκBβ and forming a positive feedback loop, promoting the pro-inflammatory actions of let-7e.

NF-κB is one of the most important regulators of inflammation. Some miRNAs, such as miR-223, miR-15a and miR-16, play crucial roles in the regulation of NF-κB activity by targeting components of the NF-κB pathway^33^,^34^,^35^. Let-7 miRNAs (let-7a, let-7f and let-7e) could increase the activation of NF-κB by targeting TNFAIP3, an inhibitor of the NF-κB pathway, and augmenting the inflammatory response in HEK293T cells as pro-inflammatory mediators^13^,^36^. The present study showed that let-7e increased NF-κB activation and played pro-inflammatory roles in endothelial cells. However, a previous study reported that let-7 acts as an anti-inflammatory mediator by targeting IL-6 and suppressing NF-κB activation in breast cells^37^. This inconsistency might primarily reflect differences in cell lines (epithelial vs. endothelial cells). Moreover, the cell- and tissue-specificity in functions and targets of miRNAs is common, and effects of the same miRNA in different cell types may be completely opposite^38^. Compared to miRNAs, studies on how lncRNAs regulate NF-κB activity have only recently emerged. It is reported that Lethe, a pseudogene lncRNA, interacts with NF-κB p65 and inhibits its activity in embryonic fibroblast cells^39^. PACER can maintain the NF-κB-dependent transcription of COX-2 in macrophages^40^.

IκBβ is one member of the IκB family, which inhibits the NF-κB pathway by masking NF-κB nuclear localization signal and sequestering it in the cytosol^41^. The pro-inflammatory genes induced through IκBα-NFκB or IκBβ-NFκB signaling and the phosphorylation and degradation pathways of IκBβ and IκBα are not exactly the same^42^. The binding of IκBβ and subsequent inhibition of NF-κB dimers are more significant in resting cells^41^. Importantly, IkBα is rapidly degraded and resynthesized after cytokine stimulation. This negative feedback limits NF-κB activation in a transient manner. However, the degradation of IκBβ is slow after cytokine stimulation and it contributes to persistent NF-κB activation^42^. This indicates that IκBβ may play more important roles in the processes of low intensity, chronic and long-term inflammation, such as atherosclerosis. Currently, studies of ncRNAs that regulate IκBβ in endothelial cells have not yet been reported. The present study is the first report that let-7e inhibits target IκBβ expression through the synergistic suppression of ceRNA lnc-MKI67IP-3. Considering the functional features of IκBβ, these results suggested that let-7e might play key roles in the chronic low-grade inflammation of endothelial cells.

In summary, the present study demonstrated that by inhibiting the target gene IκBβ, a critical inhibitor of the NF-κB pathway, let-7e promoted the nuclear translocation of NF-κB and activation, enhancing inflammatory responses in ECs. In addition, as a ceRNA, lnc-MKI67IP-3 antagonized the inhibitory effect of let-7e on IκBβ. Moreover, let-7e down-regulated lnc-MKI67IP-3, forming a positive feedback loop to aggravate inflammation (Fig. 7c). Let-7e, lnc-MKI67IP-3 and IκBβ were also abnormal in oxLDL-treated HUVECs and atherosclerotic plaques, suggesting that let-7e might play important pro-inflammatory roles in the development of atherosclerosis. The present study revealed a novel regulatory mechanism for the NF-κB pathway through ceRNA crosstalk and provided new insight into the inflammatory response of endothelial cells.

## Materials and Methods

### Ethical statement and tissue samples

Our studies have been approved by the Ethics Committee of Qilu Hospital and conformed to the Declaration of Helsinki. All coronary artery tissue samples were collected from autopsy specimens that showed atherosclerotic plaques or normality. Informed consent was obtained from all subjects (Ethic committee number KYLL-2013–038).

### Cell culture, vectors and treatment

HUVECs were purchased from American Type Culture Collection (ATCC) and cultured in recommended conditions. Cells of the same batch were used in all experiments. SiRNA and pCMV6 vectors of IκBβ and wide/mutant lnc-MKI67IP-3 were obtained from GenePharma (Shanghai, China) or Origene (Rockville, USA). The mimic and inhibitor of let-7e and corresponding negative controls (NC) were bought from GenePharma. Vitamin C was purchased from Sigma Chemical Co. (St. Louis, USA). Human ox-LDL was purchased from Yiyuan Biotechnologies (Guangzhou, China) and it was prepared as previously described^43^. Oxidation of ox-LDL was confirmed by thiobarbituric acid-reactive substances (TBARS) assay according to the manufacturer’s instructions (CellBiolabs, USA). HUVECs were treated with ox-LDL (50 υg/ml) or Vitamin C and then were harvested at 12, 24 and 48 hours after treatment. The mimic/inhibitor/NC of let-7e, siRNA or different vectors, respectively, were transfected into HUVECs using lipofectamine RNAIMAX or 3000 reagent (Invitrogen, Carlsbad, USA). HUVECs were harvested 24 hours after transfection for subsequent experiments.

### RNA isolation, Microarray and Enrichment analysis

Total RNAs were isolated from HUVECs transfected with let-7e mimic or inhibitor and corresponding NC using Trizol reagent (Invitrogen, USA) and quantified by Nanodrop 2000 spectrophotometer and Qubit 3.0 fluorometer (Thermo Scientific, USA). Their quality was also evaluated using Agilent 2100 Bioanalyzer and all RNA samples had RNA integrity numbers ≥8. Then, they were labeled with cy3 and hybridized to Agilent human LncRNA 4 × 180 K microarray (CapitalBio, China). Expression data of mRNAs and lncRNAs were filtered and normalized (ArrayExpress accession number: E-MTAB-4971), and then the lncRNAs and mRNAs regulated by let-7e were identified using GeneSpring GX 11.5 (Agilent, CA). The functional enrichment analysis was carried out using the Database for Annotation, Visualization and Integrated Discovery (DAVID) v6.7.

### Prediction of potential targets of let-7e

Let-7e targets were predicated by Targetscan, MicroT-CDS and miRanda. The transcripts, which were oppositely expressed in let-7e mimic groups and let-7e inhibitor groups, were selected from the intersection of targets predicted by the above tools and they were regarded as potential targets of let-7e in HUVECs. The MFE of heteroduplex was calculated by RNA22 v2 to filter the binding sites of let-7e in the selected transcripts (positive threshold of MFE <−12 Kcal/mol)^44^.

### Construction and analysis of co-expression/correlation network

Based on the predicted targets of let-7e, the co-expression/correlation network of lncRNAs potentially binding to let-7e and mRNAs potentially targeted by let-7e was constructed using CoExpress v1.5 (Pearson correlation coefficient >0.99, P < 0.05). The entire network was analyzed to find core subnetworks and nodes using two plugins of Cytoscape, namely ModuLand^45^ and MCODE^46^. The nodes/subnetworks in the intersection of the results of these two plugins were selected for further study as previously described^47^. The functions of lncRNAs were predicted according to their co-expressed mRNAs as previously described^48^,^49^. The functions of subnetworks were studied according to the functions of mRNAs and lncRNAs in corresponding networks and literature information.

### Real-time PCR

Total RNAs were prepared and quantified. A part of them was reverse-transcribed using Universal cDNA Synthesis Kit II (Exiqon, Denmark) for real-time PCR using LNA-enhanced let-7e family primers sets (Exiqon, Denmark) and SYBR Green master mix (Takara, Japan). A part was used for examining the pri-let-7e expression using TaqMan Pri-miRNA primers and probes (Thermo Scientific, USA). U6 was used as an internal control of miRNAs. Another part was used for real-time PCR of mRNAs/lncRNAs using PrimeScript RT reagent Kit and SYBR Green Master Mix (Takara, Japan). β-actin was used as an internal control. PCR was performed using Bio-Rad iQ5 and primers were listed in Supplementary Table S2.

### ELISA, Cell immunofluorescence and Western blot

Total, cytoplasmic and nuclear proteins were prepared using ReadyPrep Protein Extraction Kit (Bio-Rad, Hercules, CA) and aliquots of them were used for western blot analysis with antibodies to IκBβ, RELA, H3 and β-actin (Abcam, Cambridge, MA). The immunoreactive bands were detected by ECL kit and visualized with ImageQuant LAS 4000. The collected culture medium was used for the assay of ICAM1, VCAM1, SELE, SELP and IL-6 by ELISA kits (Abcam, Inc.). HUVECs were cultured on slides in 6-wells plates and transfected with let-7e mimic and negative control as above mentioned. Then, cell immunofluorescence assay was performed using anti-NF-κB mouse monoclonal primary antibody (Cell Signaling Technology, USA) and Alexa Fluor 594-labelled goat anti-mouse IgG secondary antibody (Jackson, US) as described previously^50^. DAPI (6-diamidino-2- phenylindole, Sigma-Aldrich, USA) was used to stain the nuclear. The slides were observed under LSM 710 laser confocal microscope and analyzed using ZEN software (Zeiss, Germany).

### Luciferase reporter assay, cell adhesion assay and cell apoptosis/proliferation assay

The luciferase reporter vectors were constructed by cloning the target/binding sequence (or their mutants) of let-7e in IκBβ 3′-UTR or lnc-MKI67IP-3 into pmirGLO Dual-Luciferase miRNA target expression vector (Promega, USA). HUVECs, respectively, were transfected with these vectors and let-7e mimic/inhibitor or corresponding NC. The firefly and renilla luciferase activity of cell lysates was assayed using Dual-Luciferase Reporter Assay System. The relative luciferase activity of each group was calculated according to the manufacturer’s instructions. The adhesion of monocyte (THP-1) to endothelial cell (HUVECs) was assayed as described previously^51^. The apoptosis was analyzed using Annexin V apoptosis detection kit (BD Biosciences, USA). The cell proliferation was assayed using the viable cell count method with staining by Calcein-AM (Sigma-Aldrich, USA).

### Statistical Analysis

P values in multiple testing were calibrated using Benjamini–Hochberg correction. Person correlation coefficients and p values were calculated using house-made scripts. Data were presented as mean + SEM and compared using two-tailed Student’s t-tests or one-way analysis of variance with appropriate post-tests. P < 0.05 was considered statistically significant. R package was used for all calculations. All experiments in this study were repeated at least three times independently.

## Additional Information

**How to cite this article**: Lin, Z. *et al*. Let-7e modulates the inflammatory response in vascular endothelial cells through ceRNA crosstalk. *Sci. Rep.* **7**, 42498; doi: 10.1038/srep42498 (2017).

**Publisher's note:** Springer Nature remains neutral with regard to jurisdictional claims in published maps and institutional affiliations.

## Supplementary Material

Supplementary Tables and Figures
