# Supplementary material for: Let-7e modulates the inflammatory response in vascular endothelial cells through ceRNA crosstalk
Source: Sci Rep. 2017 Feb 14;7:42498. doi: 10.1038/srep42498 (PMC5307339; doi:10.1038/srep42498)

Supplementary Information

Let-7e modulates the inflammatory response in vascular endothelial cells through ceRNA crosstalk

Zongwei Lin[1], JunfengGe[4], Zhe Wang[3], Jianwei Ren[5], Xiaowei Wang[1], Hui Xiong[6], Jing Gao[1], Yan Zhang[2,*], Qunye Zhang[1,*]

[1] The Key Laboratory of Cardiovascular Remodeling and Function Research, Chinese Ministry of Education and Ministry of Public Health; The State and Shandong Province Joint Key Laboratory of Translational Cardiovascular Medicine; Qilu Hospital, Shandong University, Jinan, China

[2] Department of Pharmacology, Shandong University School of Medicine, Jinan, China

[3] Division of Endocrinology and Metabolism, Shandong Provincial Hospital affiliated to Shandong University, Jinan, China

[4] The Second People's Hospital of Jinan, Jinan, China

[5] Health Division of Guard Bureau, General Staff Department of Chinese PLA, Beijing, China

[6] Shandong Cancer Hospital Affiliated to Shandong University, Shandong Academy of Medical Sciences, Jinan, China

Correspondence and requests for materials should be addressed to Qunye Zhang (wz.zhangqy@gmail.com) or Yan Zhang (uniquezy@163.com).

Supplementary Tables

Supplementary Table S1. The correlation coefficients (p value) between the nodes and let-7e mimic/inhibitor treatment and binding energies to let-7e

Nodes	R mimic	p mimic	R inhibitor	p inhibitor	MFE(kcal/mol)
ABCF2	-0.8884	0.3	0.9743	0.14	-15.3
CCR5	-0.9627	0.17	0.9981	0.039	-15.1
CHEK2	-0.8413	0.4	0.9926	0.08	-12.6
EFNA3	-0.9693	0.16	0.9966	0.05	-16.1
EIF3L	-0.8551	0.35	0.9456	0.21	-17.7
EIF4G1	-0.8693	0.33	0.9722	0.15	-12.4
F0X03A	-0.9799	0.13	0.9723	0.15	-20
FBRS	-0.9872	0.06	0.8991	0.29	-17.5
FURIN	-0.9208	0.26	0.9958	0.06	-15.8
G6PC3	-0.8713	0.33	0.9487	0.2	-15.1
KIFC3	-0.8757	0.32	0.9839	0.11	-13.8
lnc-MKI67IP-3	-0.9983	0.03	0.9996	0.02	-17.8
LRRC8A	-0.9507	0.2	0.8886	0.3	-12
MFSD2A	-0.9249	0.25	0.9793	0.13	-10.9
MGRN1	-0.9427	0.22	0.9975	0.04	-17.9
NFKBIB	-0.9991	0.02	0.9999	0.01	-15.6
PCNX3	-0.9609	0.18	0.9913	0.052	-18.7
PHC2	-0.8946	0.29	0.9713	0.15	-15.3
PSENEN	-0.9129	0.27	0.8778	0.32	-13.3
PSMB2	-0.8586	0.34	0.9352	0.23	-14.1
RGL2	-0.9116	0.27	0.9609	0.18	-17.6
VMA21	-0.9316	0.24	0.9909	0.087	-21.5
ZNF839	-0.9516	0.2	0.9796	0.13	-22.8
RNA41100	-0.8786	0.35	0.8911	0.31	-19.1
RNA61516	-0.8698	0.18	0.9428	0.07	-13.4

RNA41079	-0.9025	0.13	0.8677	0.17	-17.2
KLK12	-0.9501	0.06	0.9043	0.12	-11.9
CGREF1	-0.8791	0.16	0.9139	0.11	-10.4

Supplementary Table S2. The primers used in this study.

Gene	Forward (5' -> 3')	Reverse (5' -> 3')
IL-1β	AAGCTGATGGCCCTAAACAG	CTCGTTATCCCATGTGTCGA
IL-6	CACACAGACAGCCACTCACC	CACCAGGCAAGTCTCCTCAT
CCL2	CAGCCAGATGCAATCAATGCC	TGGAATCCTGAACCCACTTCT
ICAM1	ATGCCCAGACATCTGTGTCC	GGGGTCTCTATGCCCAACAA
VCAM1	TTTGACAGGCTGGAGATAGACT	TCAATGTGTAATTTAGCTCGGCA
SELE	AGAGTGGAGCCTGGTCTTACA	CCTTTGCTGACAATAAGCACTGG
SELP	CTGTTACCCTGGATTCTATGGGC	GCTGCACTGCGAGTTAAAAGA
β-actin	CACTGTGTTGGCGTACAGGT	TCATCACCATTGGCAATGAG
IκBβ	GCTGACCTTGACAAACCGGA	GCCGGATTTCTCGTCCTCG
RELA	ATGTGGAGATCATTGAGCAGC	CCTGGTCCTGTGTAGCCATT
lnc-MKI67IP-3	TGCCCGTGAATCCCTAACAG	ATGAGGGACGCATTCAGGTG
CCR5	TTCTGGGCTCCCTACAACATT	TTGGTCCAACCTGTTAGAGCTA
FOXO3A	TCACGCACCAATTCTAACGC	CACGGCTTGCTTACTGAAGG

Supplementary figures

Supplementary figure S1. (a) Purified RNA samples from HUVECs with different treatments were quantified by Qubit 3.0, a sensitive, specific and accurate fluorometer. The amounts of RNA templates for real-time PCR were ensured to be consistent in all samples. The results showed that there was no significant difference in U6 expression in HUVECs with different treatments. This justified the use of U6 as an internal control for miRNA qPCR. **(b)** Estimation of the amount of let-7e in HUVECs transfected by let-7e mimic and inhibitor. After 24 hours of transfection, the amount of let-7e was dramatically increased in HUVECs transfected with let-7e mimic and decreased in HUVECs transfected with let-7e inhibitor. However, it did not change significantly in HUVECs transfected with negative controls (NC). The untreated HUVECs were used as control. * $P<0.001$ vs. all other groups. **(c)** HUVECs were transfected by let-7e mimic and inhibitor for 24 hours. Then, the changes in expression of other ten let-7 family members was detected. The results showed no significant change in the expression of these let-7 family members, either overexpressing or inhibiting let-7e expression. **(d)** The expressions of CCR5, FOXO3A, lnc-MK167IP and NFKBIB in endothelial cells were assayed using real-time PCR. Then, the expression ratios of CCR5 (or FOXO3A) compared to lnc-MK167IP and NFKBIB were calculated and presented. **(e)** To confirm the nuclear translocation of NF-kB induced by let-7e, the immunofluorescence assays were performed on HUVECs transfected with let-7e mimic or its negative control using laser scanning confocal microscope. The images showed that let-7e significantly increased the nuclear translocation of NF-kB compared to its negative control. All experiments were repeated four times independently.

Supplementary figure S2. (a) HUVECs were treated with 200μM, 2mM and 20mM vitamin C (VitC) for 24 hours. Then, the lnc-MKI67IP-3 expression was detected using real-time PCR. The result showed that lnc-MKI67IP-3 expression was significantly induced by vitamin C. * $P<0.05$ vs. all other groups. **(b)** and **(c)** HUVECs were treated with the let-7e mimic and let-7e inhibitor at different concentrations for 24 hours. Then, the direct effect of let-7e, namely inhibiting the expression of lnc-MKI67IP-3 (b) and the target gene IκBβ (c), were detected. The results showed that at an appropriate concentration, the let-7e inhibitor counteracted the direct action of the let-7e mimic, and at high concentration, let-7e inhibitor could completely reverse the effect of let-7e. The reason the let-7e inhibitor counteracts but does not reverse the effects of let-7e is because the dose or concentration of the let-7e inhibitor was not sufficient. * $P<0.05$ vs. all other groups; ▲ $P<0.05$ vs. all other groups except for 50nM mimic+100nM inhibitor group; ◆ $P<0.05$ vs. all other groups except for 50nM inhibitor group.

Supplementary figure S3. The image of full-length immunoblots shown in figure 4b.

Supplementary figure S4. The image of full-length immunoblots shown in figure 4e.

Supplementary figure S5. The image of full-length immunoblots shown in figure 6b.

Supplementary figure S6. (a) Ox-LDL oxidation was detected by thiobarbituric acid-reactive substances (TBARS) assay. The amount of TBARS in LDL oxidized by copper was

significantly higher than that in naïve LDL. * P<0.001 vs. LDL group. **(b)** HUVECs were harvested at 12, 24 and 48 hours after treatment with ox-LDL (50 μg/ml). Then, the pri-let-7e expression was detected using real-time PCR. The untreated HUVECs were used as control. * P<0.05 vs. all other groups. ◆ P<0.05 vs. control and 12 hours groups. HUVECs were transfected by let-7e mimic and inhibitor with or without ox-LDL treatment. After 12, 24 and 48 hours, the cells were harvested for apoptosis or proliferation assays. **(c)** In HUVECs without ox-LDL treatment, let-7e mimic only slightly increased apoptosis and let-7e inhibitor insignificantly decreased it. ◆ P<0.05 vs. 0 hour groups. **(d)** In HUVECs treated with ox-LDL, let-7e mimic significantly augmented the apoptosis induced by ox-LDL, while let-7e inhibitor acted conversely. * P<0.05 vs. all other groups. **(e)** In HUVECs without ox-LDL treatment, the effects of let-7e overexpression and downregulation on cell proliferation were slight and insignificant. ★ P<0.05 vs. groups of 0 and 12 hours. **(f)** In HUVECs treated with ox-LDL, let-7e mimic markedly augmented the proliferation inhibition induced by ox-LDL, while let-7e inhibitor had the opposite effects. * P<0.05 vs. all other groups. All experiments were repeated four times independently.

Supplementary figure S7. The effect of let-7e on the endothelial inflammation induced by Ox-LDL. (a-d) HUVECs were respectively treated as shown in the figure for 24 hours. Subsequently, the expression of several inflammatory-related molecules (ICAM-1, VCAM1, SELE and IL-6) was assayed. The results showed that ox-LDL considerably induced these molecules secretion in HUVECs. Let-7e mimic further significantly increased these effects of ox-LDL, while the actions of let-7e inhibitor were opposite. The untreated HUVECs were used

as control. * P<0.05 vs. all other groups; ★ P<0.05 vs. all other groups except for controls. **(e)** Ox-LDL markedly promoted the adhesion of monocyte (THP-1) to endothelial cell (HUVEC). Let-7e mimic further significantly increased this effect of ox-LDL, while let-7e inhibitor acted conversely. ▲ P<0.05 vs. all other groups except for ox-LDL+let-7e inhibitor group. ◆ P<0.05 vs. untreated groups, ox-LDL+let-7e mimic and ox-LDL+let-7e inhibitor. * P<0.05 vs. all other groups. ★ P<0.05 vs. all other groups except for the untreated group. All experiments were repeated four times independently.

Supplementary Figure S1

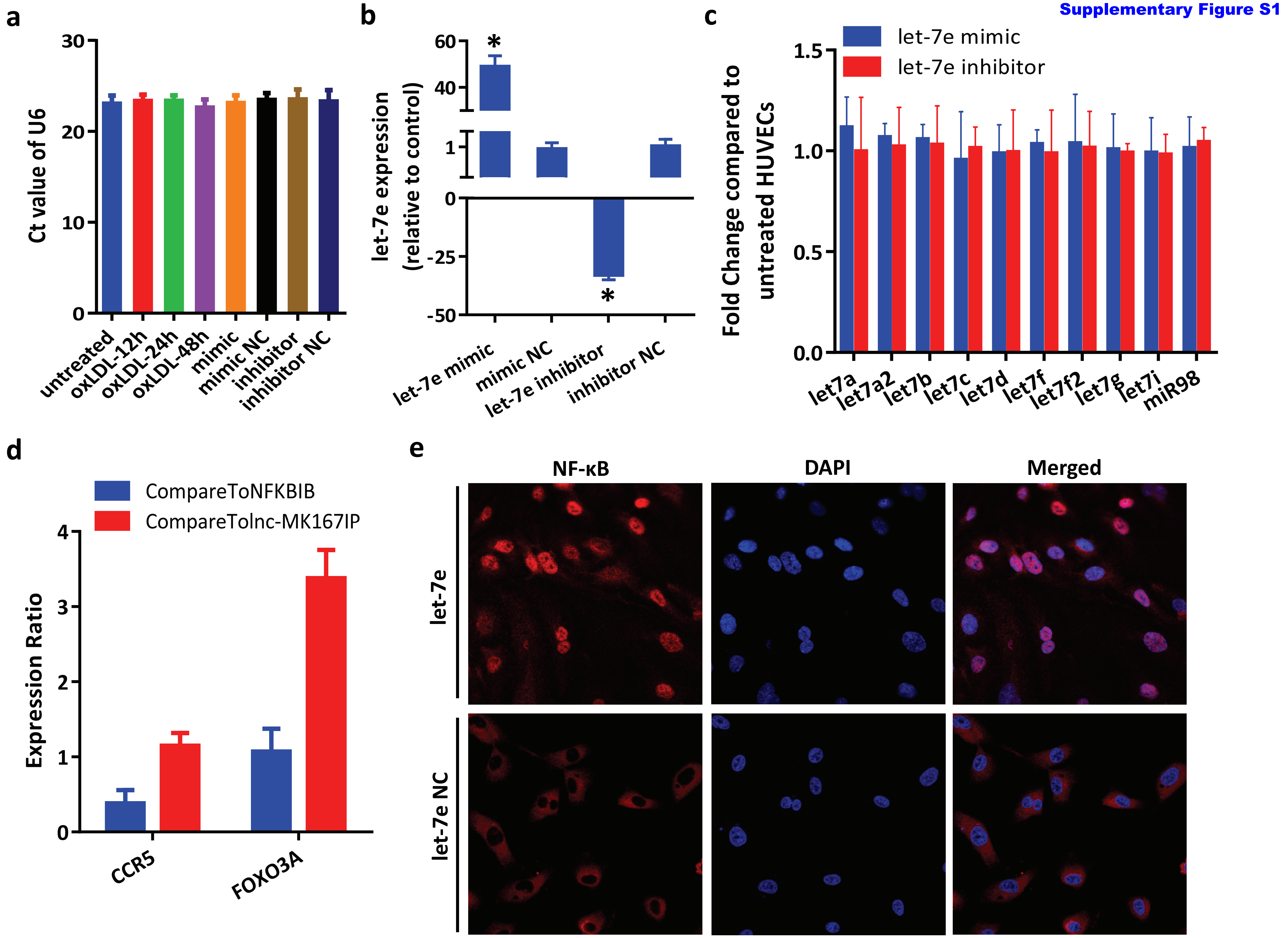

Supplementary Figure S2

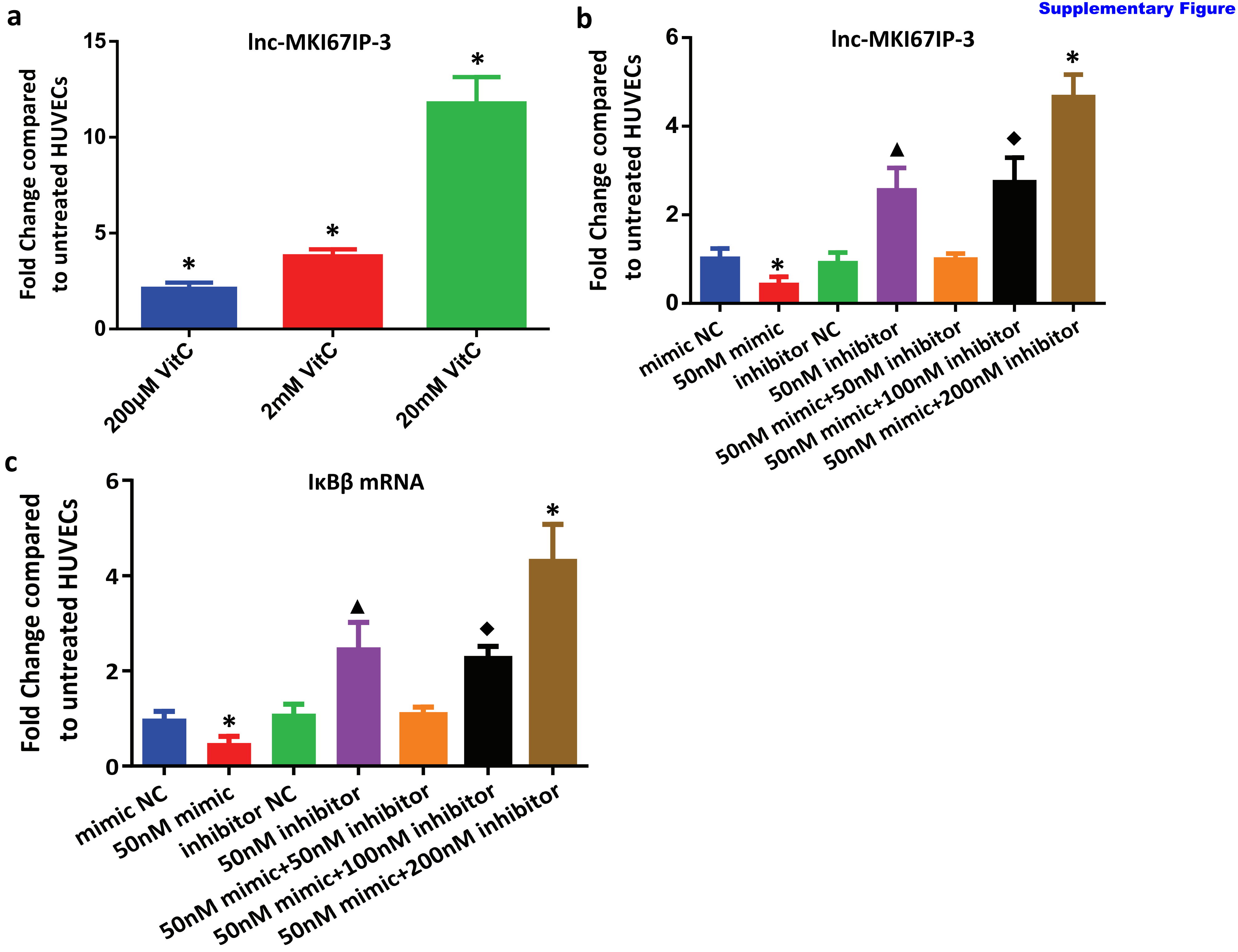

Supplementary Figure S3

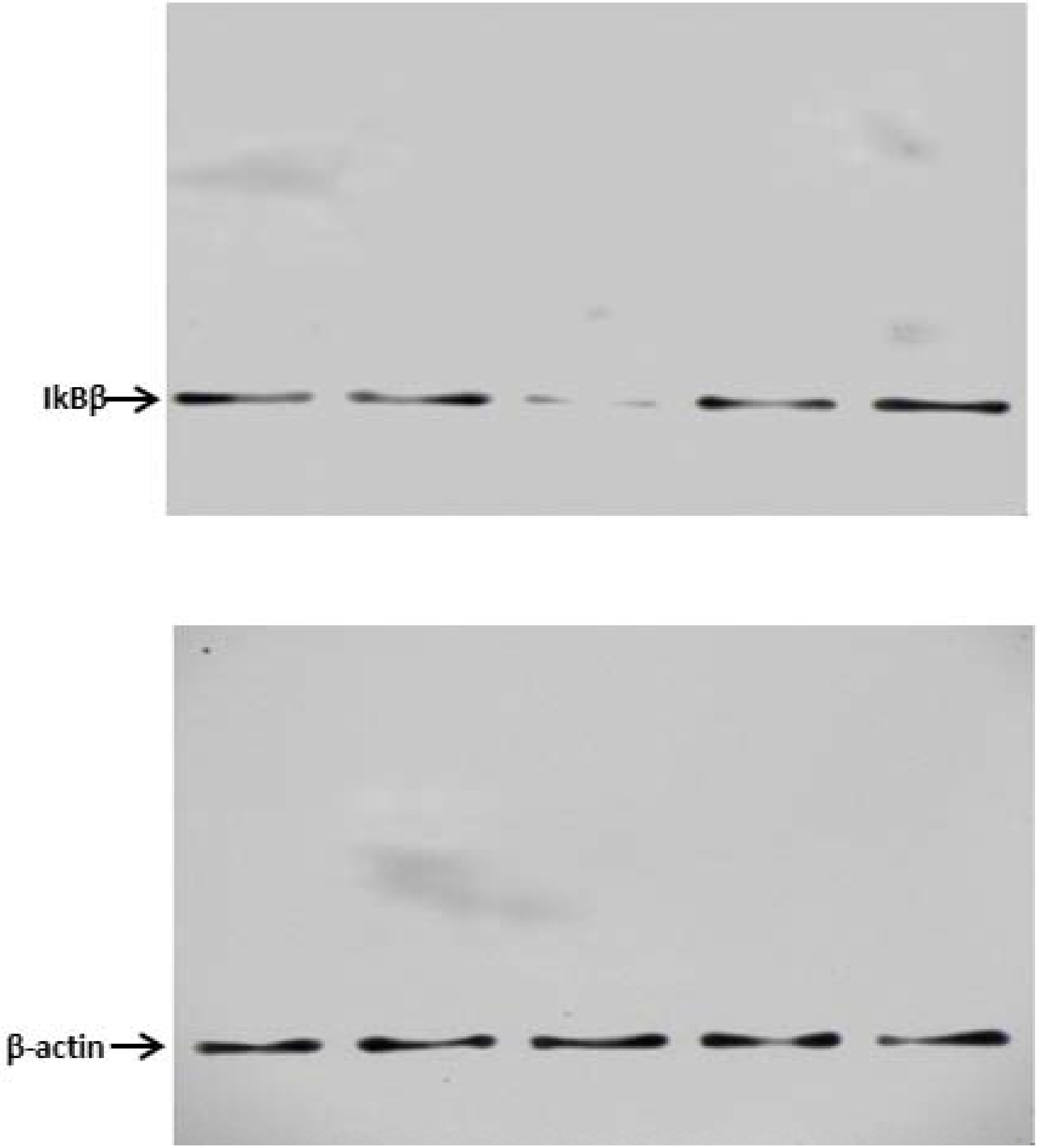

Supplementary Figure S4

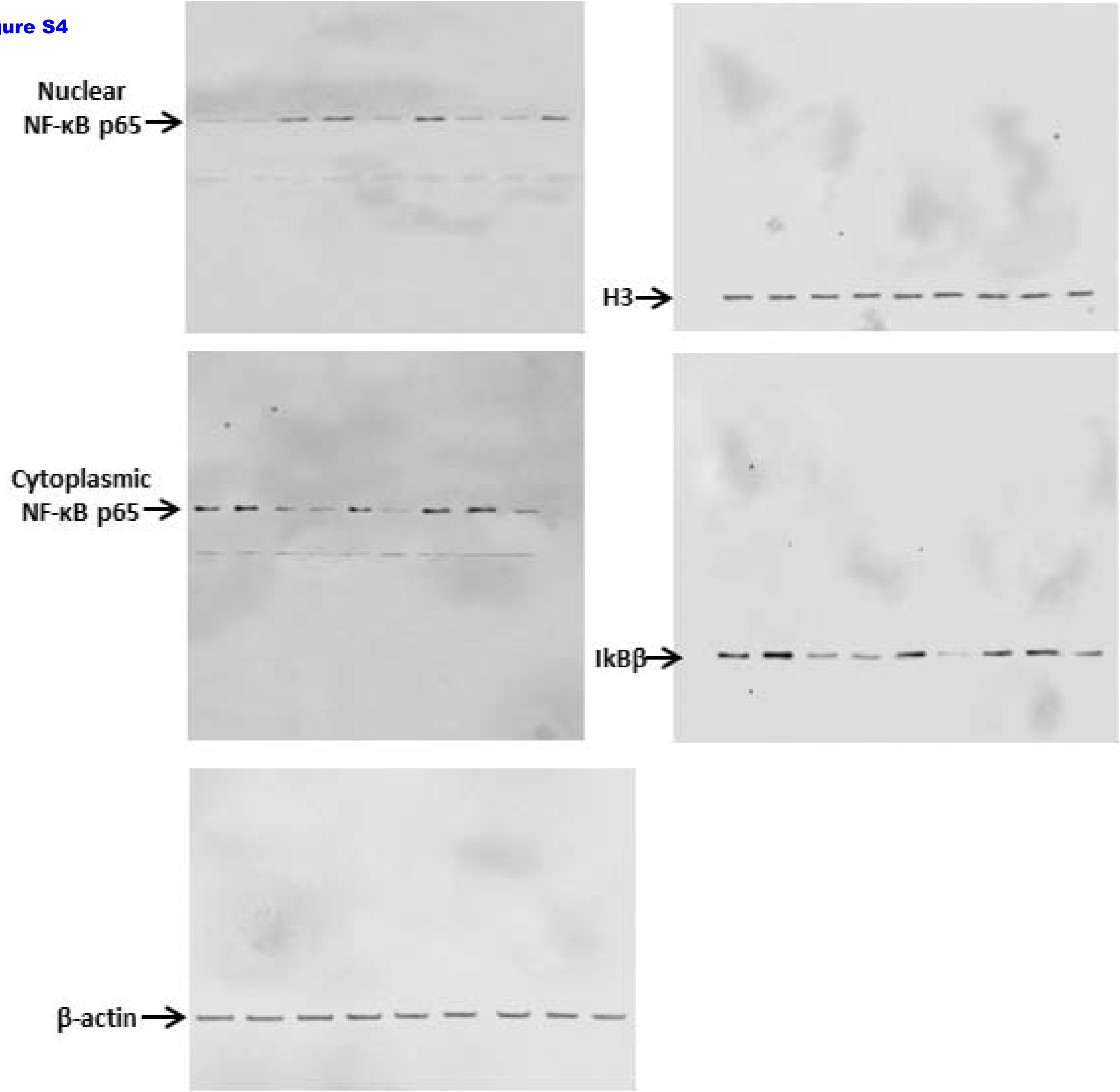

Supplementary Figure S5

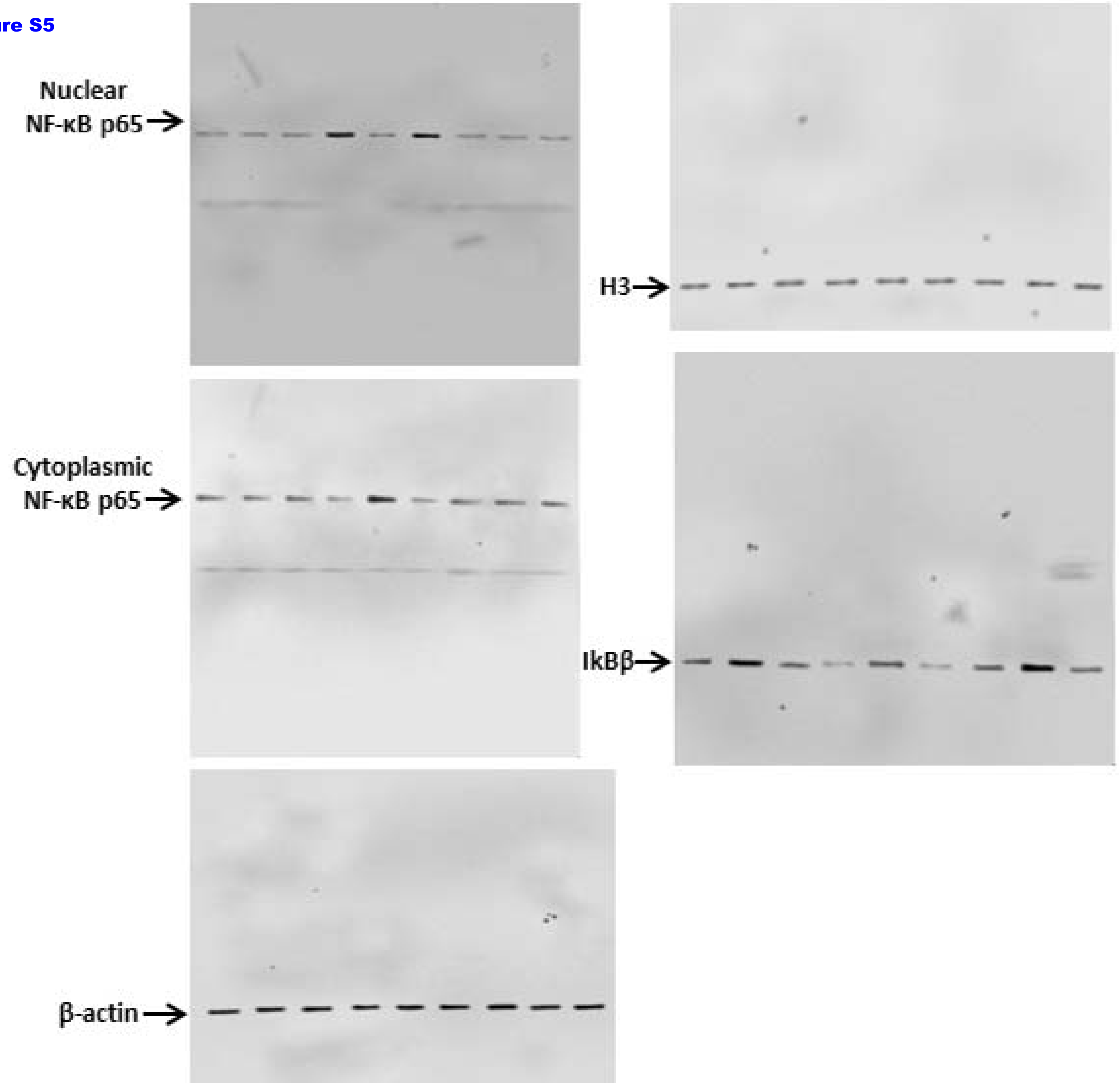

Supplementary Figure S6

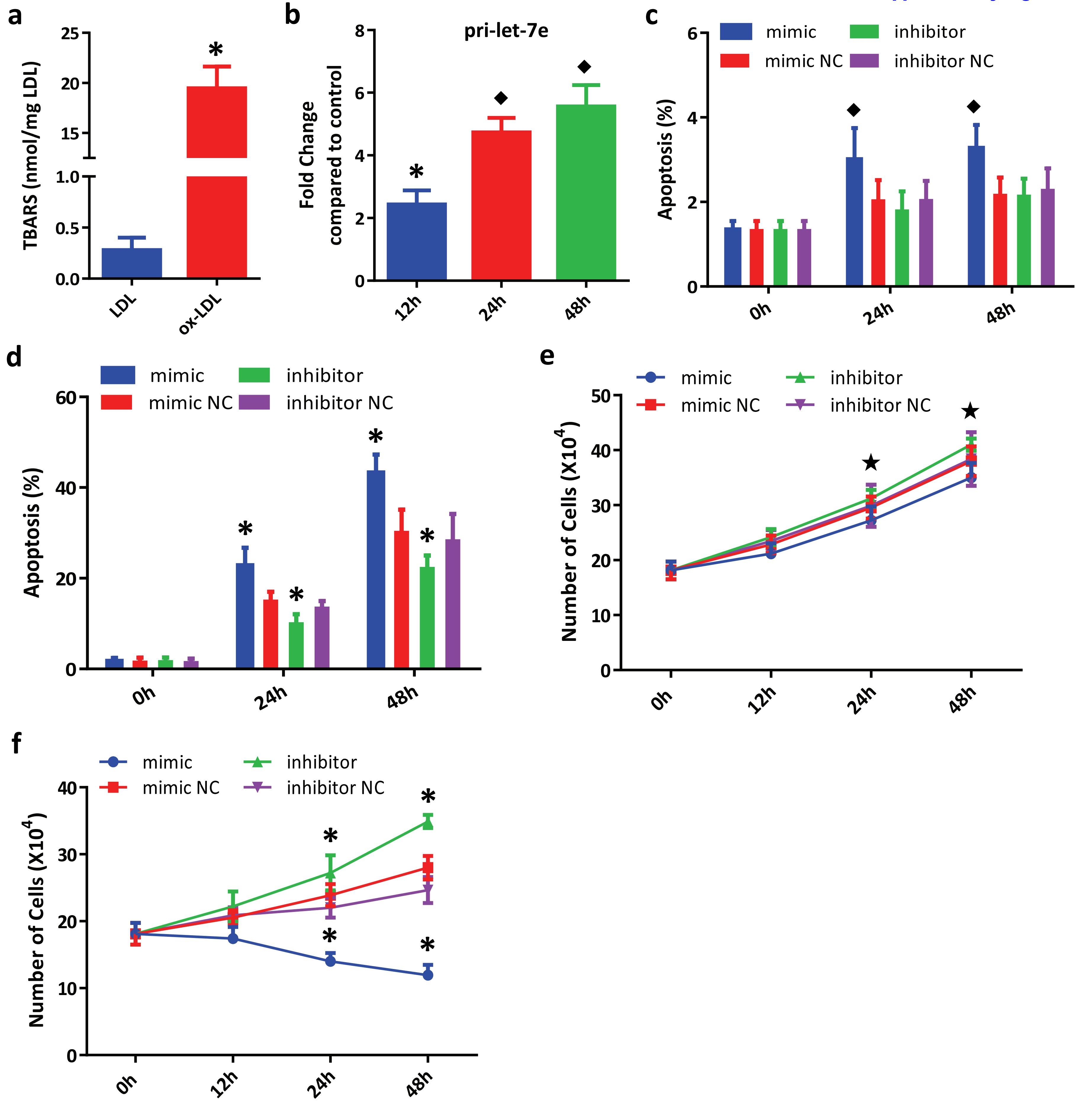

Supplementary Figure S7

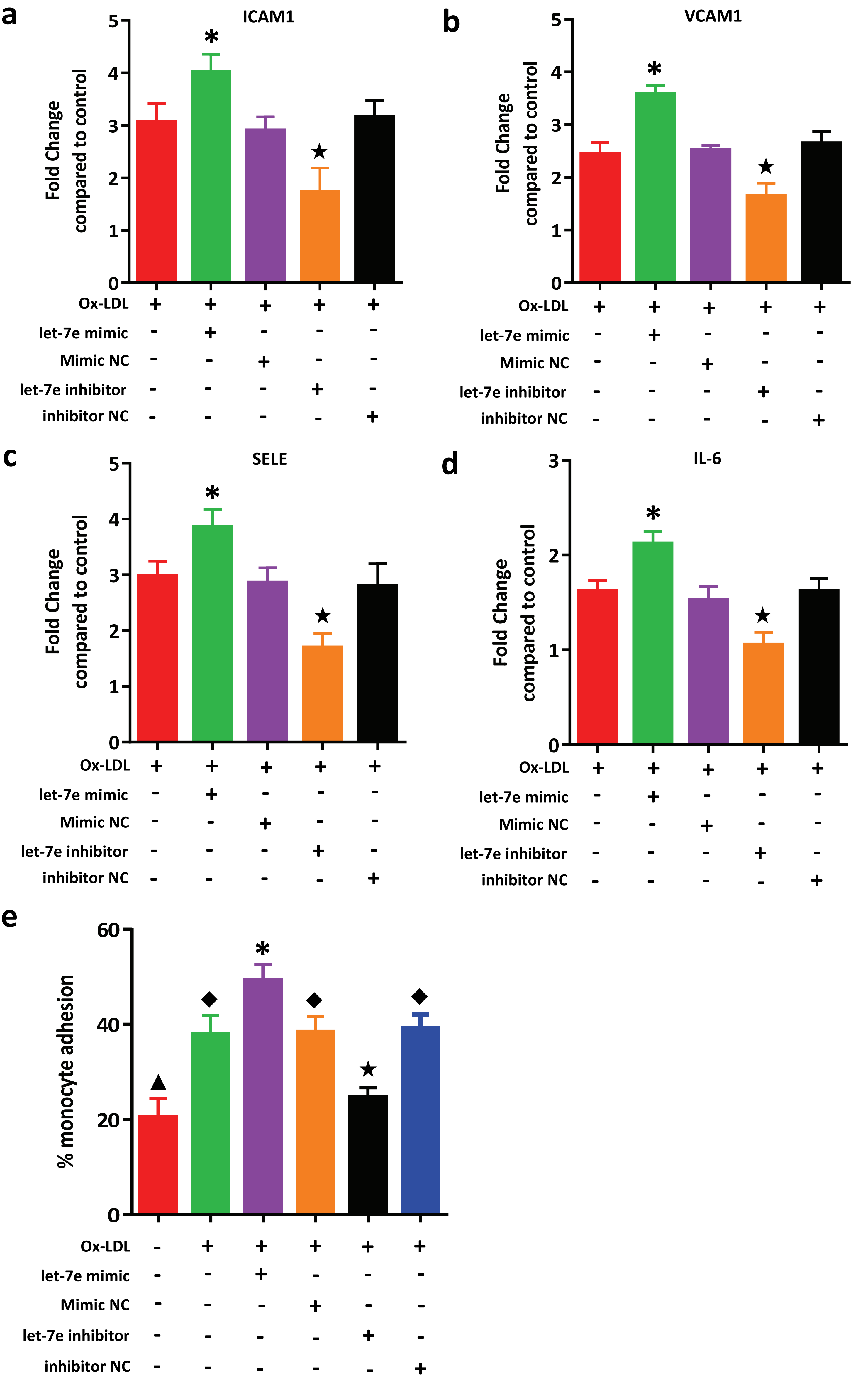

Supplement: Supplementary Tables and Figures [file srep42498-s1.pdf]
